# LEM-3 – A LEM Domain Containing Nuclease Involved in the DNA Damage Response in *C. elegans*

**DOI:** 10.1371/journal.pone.0024555

**Published:** 2012-02-23

**Authors:** Christina M. Dittrich, Katja Kratz, Ataman Sendoel, Yosef Gruenbaum, Josef Jiricny, Michael O. Hengartner

**Affiliations:** 1 Institute of Molecular Life Sciences, University of Zurich, Zurich, Switzerland; 2 Molecular Life Sciences PhD program, University of Zurich and ETH Zurich, Zurich, Switzerland; 3 Institute of Molecular Cancer Research, University of Zurich, Zurich, Switzerland; 4 Department of Genetics, The Institute of Life Sciences, The Hebrew University of Jerusalem, Jerusalem, Israel; 5 Department of Biology, ETH Zurich, Zurich, Switzerland; Duke University Medical Center, United States of America

## Abstract

The small nematode *Caenorhabditis elegans* displays a spectrum of DNA damage responses similar to humans. In order to identify new DNA damage response genes, we isolated in a forward genetic screen 14 new mutations conferring hypersensitivity to ionizing radiation. We present here our characterization of *lem-3*, one of the genes identified in this screen. LEM-3 contains a LEM domain and a GIY nuclease domain. We confirm that LEM-3 has DNase activity *in vitro*. *lem-3(lf)* mutants are hypersensitive to various types of DNA damage, including ionizing radiation, UV-C light and crosslinking agents. Embryos from irradiated *lem-3* hermaphrodites displayed severe defects during cell division, including chromosome mis-segregation and anaphase bridges. The mitotic defects observed in irradiated *lem-3* mutant embryos are similar to those found in *baf-1* (barrier-to-autointegration factor) mutants. The *baf-1* gene codes for an essential and highly conserved protein known to interact with the other two *C. elegans* LEM domain proteins, LEM-2 and EMR-1. We show that *baf-1*, *lem-2*, and *emr-1* mutants are also hypersensitive to DNA damage and that loss of *lem-3* sensitizes *baf-1* mutants even in the absence of DNA damage. Our data suggest that BAF-1, together with the LEM domain proteins, plays an important role following DNA damage – possibly by promoting the reorganization of damaged chromatin.

## Introduction

Living organisms are continuously exposed to genotoxic stress, caused both endogenously by cellular metabolism and exogenously by e.g. UV light. To maintain genome integrity, cells possess a number of DNA damage response pathways, which ensure that lesions are removed from the DNA or that cells with irreparable damage are removed by apoptosis. Defects in DNA damage responses can lead to a number of diseases, including predisposition to cancer (for review see [1]).

We and others have shown that *Caenorhabditis elegans* displays a spectrum of DNA damage responses comparable to humans, and that it can be used with success to genetically and molecularly dissect DNA damage response pathways (for review see [2]–[4]). DNA damage results in two types of cellular responses in the adult *C. elegans* germ line: In the mitotically dividing germ stem cell compartment, DNA damage results in cell cycle arrest, whereas meiotic cells in the pachytene zone respond by activating apoptosis [5]. Both responses are induced via evolutionarily conserved signalling pathways (for review see [2]–[4]). Mutants in these pathways are often characterized by defects in either or both cell cycle arrest and apoptosis.

Failure to sense or signal DNA damage can also lead to defects in DNA repair. Indeed, many *C. elegans* DNA damage response mutants display a high level of embryonic lethality following DNA damage (for review see [3], [4]), likely due to defects in the replication and segregation of the damaged genome during the rapid early cell cycles (in contrast to dividing germ cells, early embryos do not undergo cell cycle arrest following DNA damage [6]).

In 1982, Hartman and Herman took advantage of this hypersensitivity phenotype to search for genes involved in DNA damage responses. In a screen for mutants which displayed high embryonic lethality upon treatment with UV-C light, they found nine non-allelic radiation-sensitive mutants named *rad-1* to *rad-9*. For example, *rad-3* corresponds to the *C. elegans* XPA homologue *xpa-1*, which is required to remove UV-induced 6-4 photoproducts and cyclobutane dimers [7]–[9]. *rad-5/clk-2*, another mutant identified in this screen, is sensitive to various DNA damaging agents and displays defects in cell cycle arrest and apoptosis [10]. Recent studies show that the role of *clk-2* is also conserved in humans, where HCLK2 plays a critical role in the S-phase checkpoint [11], [12]. Taken together, these examples emphasize that forward genetic screens are powerful tools for identifying new key players in the complex processes following DNA damage.

As the original screen by Hartman and Herman [7] was not saturated, we conducted a similar screen for mutants displaying reduced survival upon X-radiation. Here we describe the identification and characterization of the novel evolutionarily conserved DNA damage response gene *lem-3*. LEM-3 contains a LEM (LAP2 – emerin - MAN1) domain and a GIY-YIG nuclease domain. Biochemical analysis confirms that LEM-3 acts as nuclease *in vitro*. Loss of *lem-3* function does not impair cell cycle arrest or apoptosis in the germ line but leads to high embryonic lethality following treatment with X-rays, UV-C light and cisplatin. Following DNA damage *lem-3* embryos display severe defects during cell division, including chromosome mis-segregation and anaphase bridges. These mitotic defects are similar to those found in *baf-1* (barrier-to-autointegration factor) mutants [13]. The *baf-1* gene encodes an essential and highly conserved protein known to interact with the other two *C. elegans* LEM domain proteins, LEM-2 and EMR-1 [14]–[18]. Here we show that *baf-1*, *lem-2*, and *emr-1* mutants are also hypersensitive to DNA damage and that loss of *lem-3* sensitizes *baf-1* mutants even in the absence of DNA damage. Our data uncover a novel function for *baf-1* following DNA damage, which is dependent on the LEM domain proteins.

## Results

### A forward genetic screen for mutants hypersensitive to DNA damage

**Figure 1:**
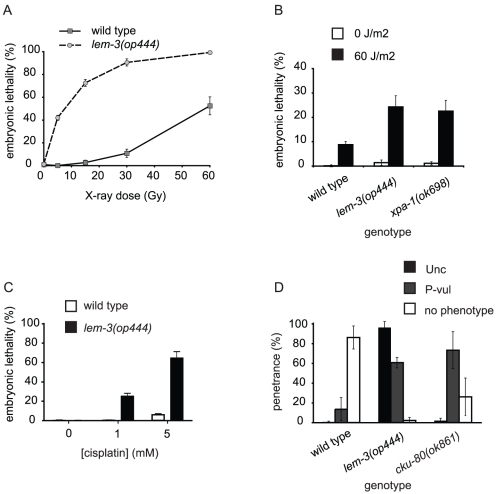
*lem-3(op444)* phenotypes following DNA damage. Wild-type animals and *lem-3(op444)* mutants were treated 24 h post L4/adult molt with (A) X-rays (B) UV-C, or (C) as L4 larvae with cisplatin. F1 embryonic lethality was assessed as described in Materials and Methods. Data shown represent the average of three independent experiments ± S.D. The progeny of 10 worms (A, B) or 5 worms (C) were analysed for each experiment. (D) Late radiation phenotypes. L1 animals were irradiated with 60 Gy. The uncoordinated phenotype (Unc) and protruding vulva phenotype (P-vul) were scored only for those animals that reached adulthood (as described in Materials and Methods). Data shown represent the average of three independent experiments ± S.D. (n for wild type, *lem-3(op444)* and *cku-80(ok861)* are 305, 236 and 373 respectively).

To find novel components involved in DNA damage response pathways, we conducted a forward genetic screen for mutations that show increased embryonic lethality following exposure to sublethal doses of ionizing radiation. In a screen of approximately 2'000 genomes, we found 14 mutants with such a phenotype (Figure S1). Of these, we selected *op444* for further analysis because it showed the highest increase in sensitivity to ionizing radiation but had no strong defect in the absence of exogenous DNA damage. Dose-response studies showed that *op444* mutants were an order of magnitude more sensitive to ionizing radiation (IR) than wild type (LD_50_ approx. 6 Gy vs. 60 Gy, Figure 1A).

### *op444* mutants are also sensitive to UV-C and cisplatin

We next tested whether *op444* mutants showed sensitivity to other types of DNA damage. UV-C light (<280 nm) induces cyclobutane pyrimidine dimers and 6-4 photoproducts, which are mainly repaired by the nucleotide excision repair (NER) pathway. To determine whether *op444* mutants are also sensitive to UV-C light, we exposed adult hermaphrodites to 60 J/m^2^ and assessed embryonic lethality of their progeny (Figure 1B). We found that *op444* mutants displayed a high lethality following UV-C exposure, similar to that observed in *xpa-1(ok698)* mutants, which carry a deletion in the *C. elegans* homolog of human XPA, a component of the nucleotide excision repair (NER) pathway [19].

We also treated *op444* animals with cisplatin, a potent DNA damaging agent frequently used in chemotherapy, which generates intra- and interstrand DNA crosslinks. *op444* mutants displayed increased embryonic lethality at both concentrations tested (1 mM and 5 mM, Figure 1C). Taken together, these data indicate that a variety of genotoxic insults can impair the survival of *op444* mutant embryos.

### *op444* is a mutation in *lem-3*

**Figure 2:**
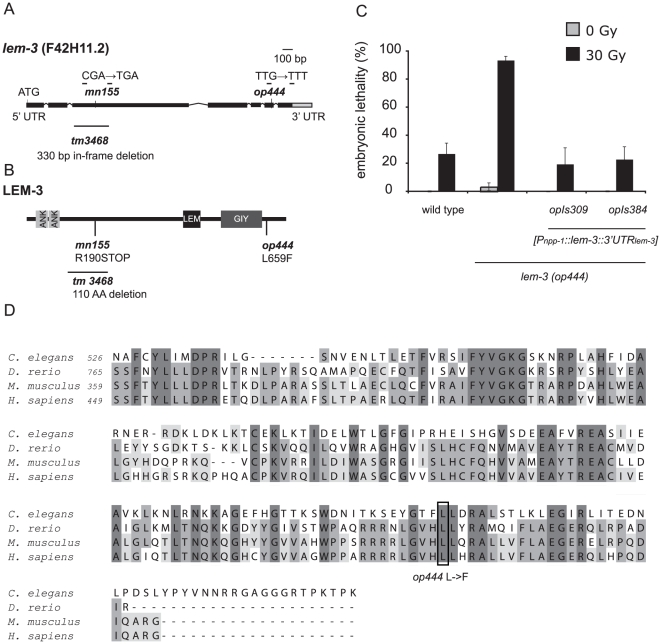
Characterization of the *lem-3* locus. (A) Gene structure of *lem-3*. Boxes represent exons, lines represent introns. The position and nature of the mutations are indicated. (B) LEM-3 is a 704 AA protein with 2 ankyrin repeats (ANK), a LEM domain (LEM) and a GIY-YIG domain (GIY) of the COG3680 type. (C) Expression of a wild-type copy of *lem-3* rescues the radiation hypersensitivity of *lem-3(op444)* mutants. Data represent the average of three experiments ± S.D. (two experiments for *opIs309*). The progeny of 10 worms were analysed for each experiment. (D) Alignment of the C-terminus of LEM-3 of different species starting with the GIY domain. The number of the first amino acid is indicated.

We took advantage of fragment length polymorphisms (FLP) and single nucleotide polymorphisms (SNP) between the Hawaii isolate CB4856 and the Bristol wild-type strain N2 [20], [21] to map *op444* to a 20 kb interval containing six predicted genes on the right arm of LG I. Unfortunately, we could not phenocopy the radiation sensitivity of *op444* mutants by RNAi knockdown of any of these six genes. However, by sequencing genomic DNA from *op444* mutants, we identified a G to T point mutation at position +2363 in *lem-3* (F42H11.2) (Figure 2A), a gene not previously implicated in any DNA damage response pathway. This mutation results in the change of an evolutionarily conserved leucine to a phenylalanine in the C-terminus of the protein (Figure 2B and D). We used biolistic transformation [22] to generate transgenic lines expressing *lem-3* under the control of the promoter of the ubiquitously expressed gene *npp-1*. Three independent transgenic lines fully rescued the radiation sensitivity of *op444* mutants; two of them are shown in Figure 2C. These results confirm that mutation of *lem-3* is the cause of the hypersensitivity phenotype observed in *op444* mutants. To determine the localisation of LEM-3, we built transgenic lines expressing N-terminally YFP tagged LEM-3. Two lines rescued the radiation sensitivity of *lem-3(op444)*, one of them is shown in Figure S2A. YFP::LEM-3 was found to be expressed only in embryos. It localised in a foci-like pattern but we could not observe it inside the nucleus (). The localisation pattern does not change following irradiation (data not shown). The exact nature of the embryonic foci is currently unclear (see discussion).

### *rad-1* mutants carry a mutation in *lem-3*

Hartman and Herman (1982) had, in a screen similar to ours, isolated and genetically characterized nine *rad* (radiation hypersensitive) genes. One of these, *rad-1(mn155)*, showed striking phenotypic similarities to *lem-3(op444)* and mapped within 2 cM of *lem-3* [23], thereby prompting us to test whether *rad-1(mn155)* is allelic to *lem-3*. Indeed, *rad-1*(*mn155*) failed to complement the radiation sensitivity phenotype of *lem-3(op444)* (Figure S3A). Sequencing revealed a C to T point mutation at position 671, which leads to a premature stop codon (Figure 2A). This R190STOP mutation leads to a truncated protein lacking the LEM domain and the GIY-YIG domain (Figure 2B and see below). Even though *rad-1* is the older name for F42H11.2, we propose *lem-3* as the common name for this gene, as the name *rad-1* has been previously used for different, non-homologous genes in various organisms.

*lem-3(op444)* also fails to complement *tm3468*, a 330 bp in-frame deletion generated by the Japanese National Bioresource Project (Figure 2A). This allele displays a weaker sensitivity to ionizing radiation (Figure S3B), possibly because the in-frame deletion does not affect any of the conserved domains of LEM-3.

### LEM-3 is a member of the GIY-YIG superfamily

The LEM-3 protein contains two ankyrin repeats in its N-terminus, followed by a LEM (LAP2 Emerin Man1) domain and a GIY-YIG domain of the type COG3680 at the C-terminus (Figure 2B and 2D). The LEM domain is an approximately 40 amino acid motif which is found in both inner nuclear membrane proteins and nucleoplasmic proteins [24], whereas the GIY-YIG domain is a nuclease domain, which is found in a diverse set of proteins that interact with DNA, including phage T4 endonuclease, restriction enzymes, and recombination and repair enzymes such as the bacterial nucleotide excision repair proteins UvrC and Cho (UvrC homologue).

### LEM-3 has nuclease activity

**Figure 3:**
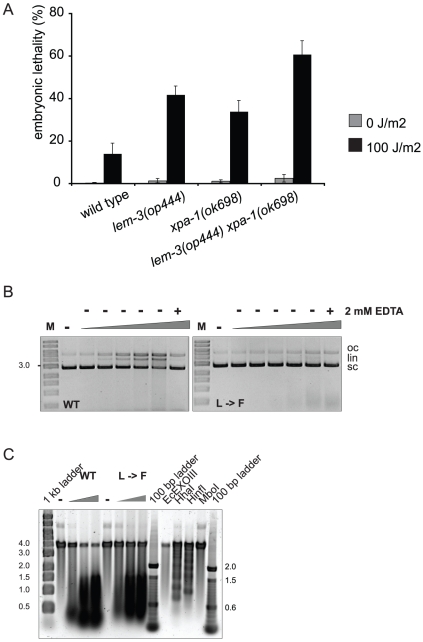
The LEM-3 endonuclease acts in parallel to the NER pathway. (A) Hermaphrodites were treated 24 h post L4/adult molt with 100 J/m^2^ and F1 embryonic lethality scored as described in Materials and Methods. Data shown represent the average of three independent experiments ± S.D. The progeny of 10 worms were analysed for each experiment. (B) Non-specific endonuclease assay, in which supercoiled plasmid DNA was incubated with increasing amounts of Sf9-expressed MBP-LEM-3 fusion protein. Endonucleolytic activity of LEM-3 (WT) leads to nicks in the DNA strands and converts the supercoiled (sc) plasmid DNA first to open-circular (oc) and then into linear (lin) DNA species. Endonucleolytic activity of LEM-3 can be inhibited by the addition of EDTA. The figure is a negative image of a 0.8% agarose gel stained with ethidium bromide. M: 1 kb marker; (−): no protein; WT: LEM-3 wild type; L→F: mutant corresponding to *lem-3(op444)*. (C) PhiX174 single-stranded DNA was incubated with increasing amounts of Sf9-expressed MBP-LEM-3 fusion protein. The single-stranded viral DNA of PhiX174 contains many secondary structures (hairpin loops, bulges etc.) that are cleaved by many structure-specific endonucleases. The figure is a negative image of a 0.8% agarose gel stained with SYBR Gold. (−): no protein; WT: LEM-3 wild type; L→F: mutant corresponding to *lem-3(op444)*. EcExoIII, *E. coli* exonuclease III; *Hha*I, *Hin*fI, *Mbo*I, restriction endonucleases.

As LEM-3 contains a GIY-YIG domain, we wanted to test whether LEM-3 also has nuclease activity. We cloned the cDNAs of wild-type *lem-3* and *lem-3(op444)* and expressed the respective proteins in insect cells (Figure S4 and Materials & Methods). Incubation of supercoiled plasmid DNA with increasing amounts of wild-type LEM-3 protein converted the supercoiled plasmid to relaxed circular (nicked) and linear molecules (Figure 3B). This reaction was dependent on the concentration of protein added and was abolished in the presence of EDTA. In contrast, addition of the mutant protein only resulted in a minor, if at all, release of the supercoiled form. To further support these findings, we investigated the enzymatic activity of LEM-3 on DNA from PhiX174, which is rich in secondary structures that can be cleaved by many structure-specific endonucleases. Incubation of wild-type LEM-3 with PhiX174 resulted in DNA cleavage (Figure 3C). Again, this endonuclease activity was significantly reduced when mutant LEM-3 was used. In conclusion, these results indicate that LEM-3 has nuclease activity and that the L to F mutation corresponding to *lem-3(op444)* greatly diminishes this activity.

### Cell cycle arrest is normal in *op444* mutants

**Figure 4:**
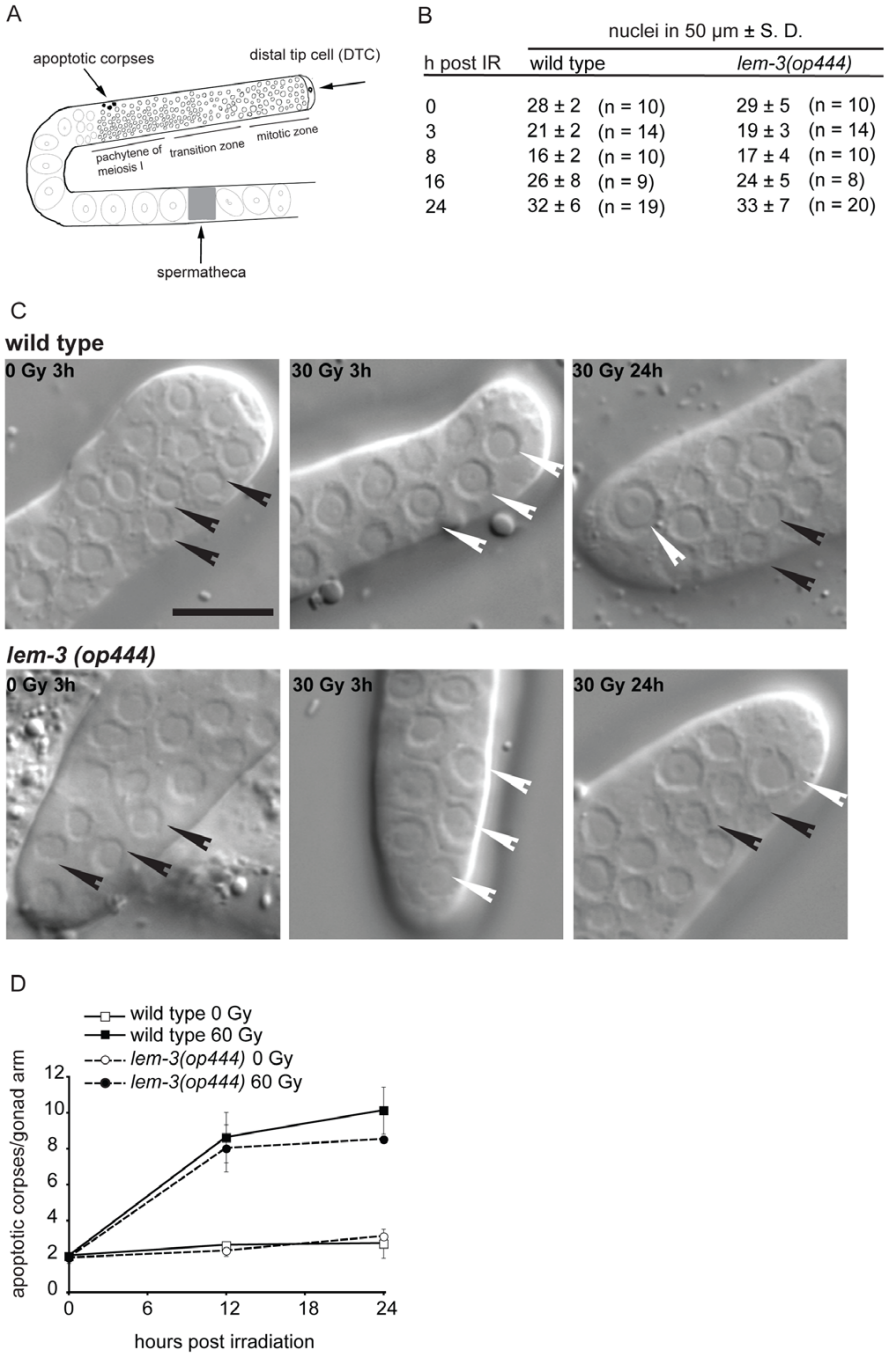
*lem-3(op444)* mutants show normal cell cycle arrest and apoptotic responses. (A) Schematic representation of a *C. elegans* gonad. The most distal part is capped by the somatic distal tip cell. The cells close to the DTC are in mitosis. As they travel down the gonad, they enter the pachytene stage of meiosis I. Near the gonadal bend, some cells die by apoptosis. Apoptotic bodies become visible as “refractive disks”. The mature oocytes are then pushed through the spermatheca and become fertilized. (B) Quantification of cell cycle arrest response following IR. The number of nuclei within 50 µm of the distant tip cell ± S.D was scored at various time points. n, number of germ lines analyzed. (C) Cell cycle arrest is normal in *lem-3*(*op444*) mutants. Representative DIC images of dissected gonads from wild-type and *lem-3(op444)* mutants are shown. Size bar: 10 µm. Black arrowheads indicate small (cycling) cell nuclei, white arrow heads indicate big (arrested) cells. (D) DNA damage-induced apoptosis is normal in *lem-3(op444)* mutants. Hermaphrodites were exposed to 60 Gy at L4/adult molt and apoptotic corpses were quantified at the indicated time points. Data shown represent the average of three independent experiments (10 germ lines per experiment) ± S.D.

DNA damage induces a transient cell cycle arrest in the mitotic stem cell compartment of the adult *C. elegans* germ line (Figure 4A), resulting in a temporary reduction in cell numbers. Because DNA damage arrests cell division but not cell growth, arrested cells eventually become much larger than in control animals (Figure 4C) [5]. Unlike many DNA damage checkpoint mutants and DNA repair mutants which are defective for cell cycle arrest [2], *lem-3(op444)* mutants showed a normal proliferation arrest and recovery following exposure to ionizing radiation (Figure 4B and C). We conclude that the pathways that sense DNA damage and signal cell cycle arrest are not impaired in *lem-3* mutants.

### Apoptotic cell death is not impaired in *op444* animals

During oocyte development (Figure 4A), approximately half of the germ cells die of apoptosis, through a developmental program called physiological germ cell death. Upon DNA damage, the number of apoptotic corpses increases significantly due to a CEP-1/p53-mediated up-regulation of the BH3 domain proteins EGL-1 and CED-13 [25]–[27]. Many DNA damage response mutants are defective in DNA damage induced germ cell apoptosis. By contrast, both basal and DNA damage induced apoptosis were normal in *lem-3(op444)* animals (Figure 4D). We conclude that regulation and execution of apoptosis is unimpaired in *lem-3(op444)* animals. Given that cell cycle arrest and apoptosis following DNA damage are both normal in *lem-3(op444)* mutants, we speculate that *lem-3(op444)* mutants are not impaired in DNA damage signalling but rather in another part of the DNA damage response system, most likely DNA repair.

### Analysis of DNA damage response in *lem-3(op444)* mutants

Based on our data above, we next asked which repair pathway LEM-3 might participate in. We first investigated double strand break repair, as this process occurs extensively in the germ line as part of meiotic recombination. Failure to resolve the double strand breaks induced by the SPO-11 endonuclease results in increased germ cell apoptosis due to activation of CEP-1/p53 and increased meiotic non-disjunction. The resulting aneuploidy leads to a high embryonic lethality and a high incidence of males. However, none of these phenotypes is apparent in *lem-3(op444)* mutants in the absence of exogenous DNA damage (Figure 1A, 4D, and data not shown). Thus, we conclude that *lem-3* mutants are proficient in both meiosis and the resolution of dsDNA breaks induced during meiotic recombination.

**Figure 5:**
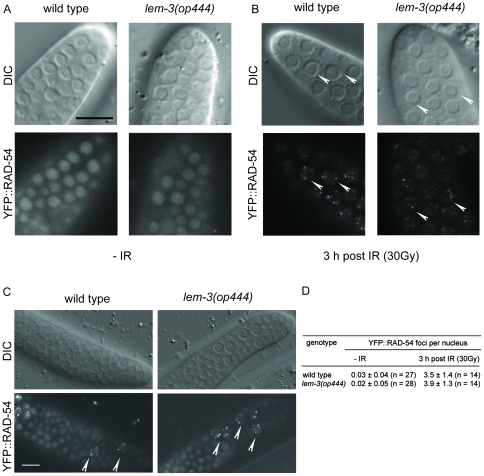
RAD-54 foci formation and removal is normal in *lem-3*(*op444*) mutants. DIC and fluorescence images of control (A) and irradiated (B) embryos expressing YFP::RAD-54(*opIs257*). RAD-54 foci (arrow heads for selected nuclei) are induced in the mitotic zone upon irradiation. (C) By 17 h post IR, foci are largely removed in both wild-type and *lem-3(op444)* animals. Size bar: 10 µm. (D) Quantification analysis of YFP::RAD-54 foci formation. n, number of germ lines analyzed.

Rad54 has been implicated in several steps during homologous recombination and repair [28]. Down-regulation of *rad-54* by RNAi results in increased apoptosis and radiation-induced embryonic lethality [29]. We have already shown that RAD-54 forms foci following IR, likely marking sites of active repair [30]. To get further insights into the ability of *lem-3(op444)* mutants to repair exogenously generated dsDNA breaks, we analyzed the subcellular localisation of YFP::RAD-54. In the absence of exogenous damage, YFP::RAD-54(*opIs257*) localized to distinct foci in the meiotic zone of the germ line, but showed a diffuse nuclear staining pattern in the mitotic zone (Figure 5A). This is in line with the known role of RAD-54 during homologous recombination. Upon irradiation, foci also appeared in the mitotic zone (Figure 5B). Quantitative analysis revealed that the number of RAD-54 foci per nucleus were similar in wild type and *lem-3(op444)* (Figure 5D). After 17 hours, most cells in the mitotic zone had successfully removed RAD-54 in both wild type and *lem-3(op444)*. Only a few, enlarged nuclei still showed RAD-54 foci (Figure 5C). These likely represent cells with persistent DNA damage that failed to re-enter the cell cycle. We conclude from these data that the repair of exogenously generated dsDNA breaks is normal *lem-3(op444)*.

### *lem-3* and *xpa-1* act in separate pathways

Since *lem-3* mutants are also hypersensitive to UV-C light, we investigated a possible involvement of *lem-3* in the NER pathway. We built and analyzed a double mutant with *xpa-1(ok698)*, a factor common to transcription-coupled repair and global genomic repair, the two branches of the NER pathway. Interestingly, we found that the double mutant showed a significant increase in embryonic lethality compared to both single mutants (Figure 3A). Thus, we conclude that *lem-3* and *xpa-1* likely do not act in a linear pathway.

### LEM-3 is required for proper chromosome segregation following DNA damage

**Figure 6:**
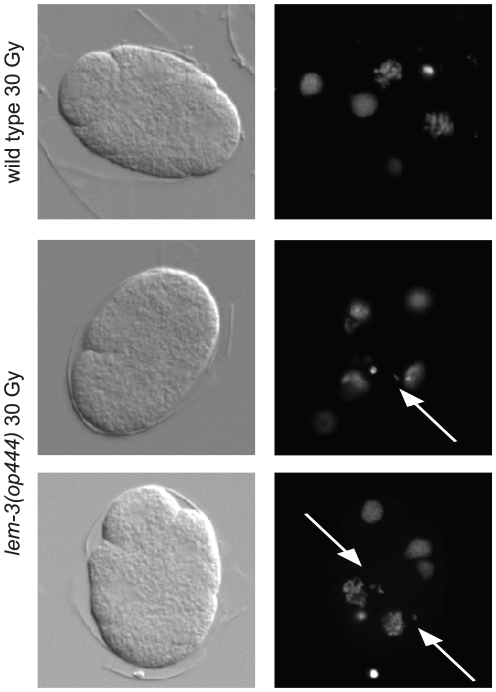
Chromatin segregation defects in *lem-3*(*op444*) embryos after irradiation. Embryos were dissected from irradiated hermaphrodites, DNA was stained with Dapi. Representative DIC and fluorescence pictures are shown. Arrows point to abnormally localized chromatin.

As we could not find any clear defects in the germ line of *lem-3(op444)* mutants, we investigated in more detail the cause of the lethality observed following DNA damage. To get a first insight into the nature of the defect, we stained chromatin in early stage embryos derived from irradiated hermaphrodite worms. Whereas chromosome segregation was mostly normal in irradiated wild type embryos (91%, n = 67), 77% (n = 31) of irradiated *lem-3(op444)* embryos showed chromosome mis-segregation starting at the second cell division, as evidenced by the generation of extra nuclear chromatin material and chromatin bridges following mitosis (Figure 6). We confirmed this result by using a GFP::H2B transgene to follow chromosome segregation in early embryos by time lapse microscopy (Movie S1 and Movie S2). To determine whether LEM-3 is also required for proper chromosome segregation during larval development, we irradiated freshly hatched L1 larvae and scored the animals as adults for locomotion defects or protruding vulvae – phenotypes arising from defective proliferation/division of the Pn.a neuroblast or Pn.p vulval precursor cells, respectively. These phenotypes can also be observed following irradiation of mutants of the non-homologous end joining pathway genes *cku-70*, *cku-80* and *lig-4* [31]. Only 2% of irradiated *lem-3* mutants developed to adults without any developmental defects (96% displayed an uncoordinated phenotype (Unc), 61% had a protruding vulva (P-vul)). In contrast, 86% of irradiated wild type L1 larvae developed to phenotypically normal adults (Figure 1D). These results suggest that *lem-3* mutant embryos die after irradiation because of loss of genomic integrity due to chromosome segregation defects. Additionally, we conclude that *lem-3* is also required during post-embryonic cell divisions after genotoxic stress to ensure normal cell proliferation.

### LEM-3 genetically interacts with BAF-1

Three LEM-domain proteins, EMR-1 (Ce-emerin), LEM-2 (Ce-MAN1), and LEM-3 have been identified in *C. elegans*. EMR-1 and LEM-2 have a transmembrane domain and an N-terminally located LEM domain [14], whereas LEM-3 lacks a transmembrane domain, and has a LEM-domain located in the middle of the protein [14]. EMR-1 and LEM-2 bind LMN-1, the *C. elegans* lamin homologue, and BAF-1 (barrier-to-autointegration factor) [15]. BAF is an evolutionarily conserved and essential protein which was shown to interact with dsDNA, chromatin, nuclear lamina proteins, histones and transcription factors [32]. Downregulation of either *lmn-1* or *baf-1* or co-depletion of *emr-1* and *lem-2* leads to severe defects in mitosis, including abnormal chromosome segregation and anaphase bridges, which ultimately leads to embryonic lethality [13], [15], [18], [33]. BAF-1 is essential for nuclear envelope formation [34] and required to assemble LMN-1, EMR-1 and LEM-2 on the nuclear envelope [17].

**Figure 7:**
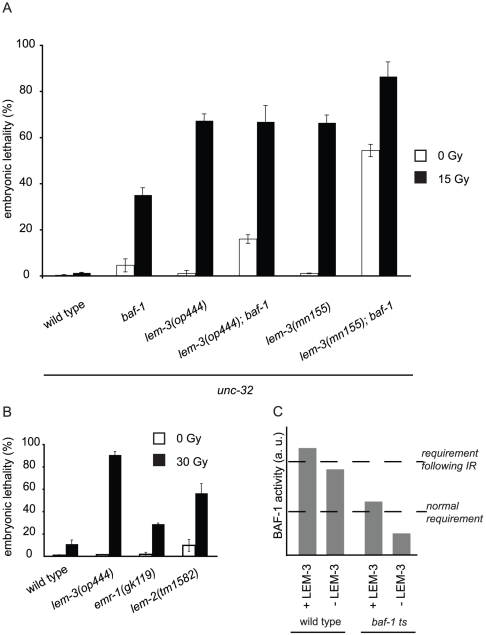
LEM-domain proteins and BAF-1 cooperate to protect embryos from DNA damage-induced lethality. (A) Hermaphrodites were treated 36 h post L4/adult molt with 15 Gy and F1 embryonic lethality was scored 10 h later as described in Materials and Methods. All assays were done on 15°C, which is the permissive temperature for *baf-1(t1639ts)*. All strains used had *unc-32(e189)* in the background. Data shown represent the average of three independent experiments ± S.D. The progeny of 10 worms were analysed for each experiment. (B) Animals were treated 24 h post L4/adult molt with 30 Gy. Data shown represent the average of three independent experiments ± S.D. The progeny of 10 worms were analysed for each experiment. (C) Model of *lem-3* and *baf-1* interaction. We propose that the requirements for BAF-1 activity are higher following DNA damage. In wild-type animals, LEM-domain proteins stimulate BAF-1 activity sufficiently to protect embryos from death. In the absence of LEM-3 this level is no longer met, and thus leads to embryonic lethality following DNA damage. In *baf-1 ts* mutants at permissive temperature, BAF-1 activity is compromised and does not meet the requirement following DNA damage, in the absence of LEM-3 it is further reduced so that it is even insufficient under normal conditions.

As downregulation of either *baf-1* or co-depletion of *emr-1* and *lem-2* leads to embryonic lethality [13], [15], [18] with phenotypes similar to those observed in *lem-3* mutants following IR, we analyzed whether *baf-1*, *lem-2* or *emr-1* mutants might be sensitive to DNA damage. We found that *emr-1* mutants were weakly sensitive, while *lem-2* mutants displayed moderate sensitivity to IR (Figure 7B). The temperature sensitive *baf-1(t1639ts)* mutant at permissive temperature was also hypersensitive to irradiation treatment (Figure 7A). To test whether *baf-1* and *lem-3* interact genetically, we built double mutants with both *lem-3* alleles and analyzed their sensitivity to irradiation. Interestingly, the complete loss of LEM-3 function (*lem-3(mn155)*) greatly increased the lethality of the *baf-1(t1639)* mutation, even in the absence of exogenous DNA damage. By contrast, the double mutant with the point mutation (*lem-3(op444)*) showed only a minor increase in lethality, likely due to residual activity of the LEM-3 (L659F) protein. This result suggests that when BAF-1 function is limiting, LEM-3 function becomes important even in the absence of DNA damage. Taken together, the phenotypes described in irradiated *lem-3* mutants and animals depleted of either *lmn-1* or *baf-1*, or co-depleted of *emr-1* and *lem-2* suggest a possible common cause of the observed molecular defects.

## Discussion

Here we report our identification of *lem-3* as a new gene required for proper DNA damage response in *C. elegans*. We isolated the *lem-3(op444)* mutation in a forward genetic screen for animals hyper-sensitive to ionizing radiation. DNA damage response mutants are often defective in DNA lesion-induced cell cycle arrest or apoptosis. As cell cycle arrest (Figure 4B and C) and apoptosis (Figure 4D) were normal in *lem-3* mutants, we conclude that, unlike HUS-1 [25], CLK-2/RAD-5 [5], [10], or CEP-1/p53 [35], [36], LEM-3 is likely not involved in the initial signalling process following DNA damage.

### LEM-3 belongs to the GIY-YIG superfamily of nucleases

Our bioinformatic analysis revealed the presence of a GIY-YIG domain in LEM-3, which can be found in various enzymes involved in DNA repair and recombination [37]. When tested in two different types of assays *in vitro*, wild-type LEM-3 was able to cleave DNA, while the activity of the L659F (*op444*) mutant was largely reduced (Figure 3B and C). Given these results, we postulate that LEM-3 is a novel endonuclease involved in DNA damage repair. The exact repair pathway (if any) LEM-3 might participate in remains however to be identified.

### LEM-3 is conserved in humans

LEM-3 is evolutionary conserved. A report by Brachner et al. (co-submitted) demonstrates that the human LEM-3 homologue Ankle1 is capable of inducing DNA cleavage when forced to localize to the nucleus. A leucine to phenylalanine mutation corresponding to the mutation in *lem-3(op444)*, however, failed to cause DNA damage under the same conditions. These observations suggest that the molecular function of LEM-3/Ankle1 might be conserved through evolution.

### Subcellular localization of LEM-3

We found that YFP::LEM-3 localizes in a distinct foci-like structure clearly outside of the nucleus (Figure S2B). To date, we have no definitive explanation for this localization pattern, which is independent of DNA damage. We speculate that LEM-3 is present at low concentrations in the nucleus where it fulfills its function. At the onset of anaphase, microtubules pull the chromosomes into a poleward direction. In between, non-kinetochore microtubles form a bundled structure and assemble as part of the central spindle complex, also referred to as the “midbody”. The observed foci could be remnants of the midbody – we were however unable to confirm this hypothesis.

### Interaction between LEM-3 and BAF-1

Whereas *lem-3* mutants do not show any defects in the adult germ line following DNA damage, embryos generated from irradiated germ lines show major defects in chromosome segregation starting at the second cell division. This phenotype is reminiscent of embryos depleted of *lmn-1*, *baf-1* or co-depleted of *lem-2* and *emr-1*. We found that a reduction of BAF-1 function in *lem-3* mutants causes high embryonic lethality even in the absence of any genotoxic insult. Our data suggest a model in which LEM-3 is required to support BAF-1 function. We postulate that, for a currently unknown reason, requirement for BAF-1 function increases following DNA damage. What exact function BAF-1 plays following DNA damage and how LEM domain proteins contribute to this function remains to be determined. Loss of *lem-3* function uncovers this requirement. This model is supported by the fact that also the other *C. elegans* LEM domain protein mutants *lem-2* and *emr-1* show a moderate and a weak sensitivity to IR, respectively.

## Materials and Methods

### Strains and general procedures

N2 Bristol strain [38] was used as the wild-type strain in all experiments. For mapping experiments the polymorphic strain CB4856 was used. All strains were kept on NGM agar plates seeded with Escherichia coli OP50 at 20°C unless otherwise stated.

#### Mutant alleles

*lem-3(op444)* (this study), *rad-1(mn155)* [7], *lem-3(tm 3468)* (this study), *rad-5*(*mn*159) [7], *xpa-1(ok698)* [19], *cku-80(ok861)* [31], *lem-2*(*tm1582*) (this study), *emr-1(gk119)* [39], *baf-1(t1639)* [40].

#### Transgenes

*opIs257* [P*_rad-54_*::*rad-54*::YFP::3′UTR*_rad-54_*] [30], *opIs309* [P*_npp-1_*::*lem-3*::3′UTR*_lem-3_*] (this study), *opIs384* [P*_npp-1_*::*lem-3*::3′UTR*_lem-3_*] (this study), *opIs383* [P*_npp-1_*::YFP::*lem-3*::3′UTR*_lem-3_*] (this study), *ruIs32* [*unc-119(+) pie-1::*GFP::H2B] [22].

### Ethyl methane sulfonate (EMS) based screen

Synchronized L4-stage wild-type worms [38] were subjected to 50 mM EMS (M0880 Sigma-Aldrich) and incubated at 20°C for 4 hours. The screening was performed according to the second scheme previously described [7]. But instead of UV-C light, a dose of 60 Gy was used for inflicting DNA damage.

### Irradiation

An Isovolt Titan 160 with Isovolt 160 M2/0.4–3.0 (Seifert) and a Stratalinker UV crosslinker, model 1800 (Stratagene) were used to deliver the indicated doses.

### Embryonic lethality (X-ray and UV-C)

Animals were irradiated 24 h post L4/adult molt, individualized on plates and allowed to lay eggs for 4 hours. Eggs were quantified; unhatched eggs were counted 24 h later, and the percentage of embryonic lethality was calculated. All assays were performed on 20°C except otherwise stated.

### Late Rad phenotypes

Animals were synchronized (freshly hatched L1s) and irradiated. Phenotypes of those animals that had reached adulthood were quantified 3 days later using a dissection microscope. The Uncoordinated (Unc) phenotype was scored on the basis of sluggish movement.

### Cisplatin treatment

For embryonic survival assays synchronized animals (L4/adult molt) were exposed to different doses of cisplatin (Sigma-Aldrich) on agar plates by evenly distributing 1.2 ml of the corresponding concentration of cisplatin on a 10 cm plate. Animals were transferred to fresh plates 24 h post-treatment. Animals were allowed to lay eggs for 12 hr. Adults were then removed and eggs were counted. Unhatched eggs were quantified 24 h later, and the percentage of embryonic lethality was calculated.

### Germline apoptosis

Staged animals (12 hours post L4/adult molt) were irradiated with 60 Gy. Apoptotic corpses were quantified in the meiotic zone of the germ line at indicated time points, as previously described [41].

### Cell cycle arrest

Staged animals (L4/adult molt) were irradiated. Germ lines were dissected at the indicated time points. Images were taken using an ORCA-ER digital CCD camera. The diameters of 10 nuclei (in focus) were measured and the average diameter of nuclei per germ line was calculated using ImageJ 1.40 g software (Wayne Rasband, http://rsb.info.nih.gov/ij).

### RAD-54::YFP foci

Staged animals (12 h post L4/adut molt were irradiated. Germ lines were dissected 3 hours after treatment. Images were acquired with a Leica DMRA2 microscope equipped with an ORCA-ER digital CCD camera. Foci were analyzed with ImageJ 1.40 g software (Wayne Rasband, http://rsb.info.nih.gov/ij).

### FM4-64 staining

Embryos were dissected from gravid adults in M9 containing 64 µM FM4-64 (Invitrogen). The eggshell was cracked as previously described [42].

### Time lapse microscopy

For time lapse microscopy, an Olympus BX61 was used equipped with a Retiga 2000R camera. Dissected embryos were put on a 2% agarose pad. Images were taken every 20 seconds at 2× binning. Pictures were processed using Openlab software (Improvision).

### Alignments

Alignments were ClustalW alignment done with the Jalview software (Version 11.0).

### Transgenic lines

Transgenic lines were obtained by microparticle bombardment in a Biolistic PDS- 1000 (Bio-Rad) transformation, as previously described [22].

### DNA constructs

PCR amplification was done with Phusion High-Fidelity DNA Polymerase (Finnzymes). Oligonucleotide primers were synthesized by Microsynth.

### Primers

*npp-1* promoter

*Pnpp-1Sbf_fw* GATCCCTGCAGGTTATTGGTGTCATTTCGGTGATTATGATTG

*Pnpp-1Asc_rev* CCATGGCGCGCCTTCGCTGAAAACAAACGATTTTTAAGAGAAATG

*lem-3* genomic plus 3′UTR

*3lem3AscIfw*

GATCGGCGCGCCATGCCTCCAAACGGAGCAATCACCACGA

*3lem3ApaIrev*

CTGAGGGCCCAGCCAATTCAACCGACTAATAAGGTAGATT

### Expression and enrichment of LEM-3

The cDNA of LEM-3 WT and L→F mutant was cloned into a modified pFastBac1 vector (a kind gift of Petr Cejka) that contains an N-terminal MBP-tag. Baculovirus stocks were generated by a procedure described earlier and Sf9 cells (400 ml, 1×10^6^ cells per ml) were infected following the User's Manual (Gibco BRL). Sf9 cells expressing LEM-3 were harvested 48 h post infection by centrifugation, washed once in PBS and snap frozen. For enrichment, the pellet was resuspended in Column-buffer (25 mM Hepes-KOH pH 7.4, 150 mM KCl, 10% glycerol, 1 mM DTT, 0.1 mM EDTA) containing 1 mM PMSF and a proteinase inhibitor cocktail (Roche), and sonicated. After centrifugation (18 000 g, 1 h, 4°C), the supernatant was incubated with Amylose resin (NEB). Subsequently, the column was washed with Column-buffer and MBP-LEM-3 was eluted (Elution buffer: 25 mM Hepes-KOH pH 7.4, 150 mM KCl, 10% glycerol, 1 mM DTT, 0.1 mM EDTA, 10 mM Maltose). Fractions containing MBP-LEM-3 were pooled, snap frozen and stored at −80°C.

### Non-specific endonuclease assay with supercoiled plasmid DNA

80 ng of supercoiled pFastBac1 plasmid were incubated with increasing amounts of LEM-3 wild type or its op444 mutant in endonuclease buffer (25 mM Hepes-KOH pH 7.4, 25 mM KCl, 1 mM MgCl_2_). After incubation at 25°C for 1 h, the reaction was terminated by the addition of 0.1% SDS, 14 mM EDTA and 0.1 mg/ml Proteinase K and incubation at 55°C for 10 min. 10% glycerol was added and the samples were separated on a 0.8% agarose gel for 45 min at 80 V. [43].

### PhiX174 non-specific endonuclease assay

PhiX174 circular virion ssDNA (NEB) was incubated with LEM-3 wild type and L→F mutant in endonuclease buffer (25 mM Hepes-KOH pH 7.4, 60 mM KCl, 1 mM MgCl_2_) for 1 h at 25°C (*EcExoIII*, *HhaI*, *HinfI* and *MboI* were incubated for 4 h at 25°C); The reaction was stopped by addition of 0.1% SDS, 14 mM EDTA and 0.1 mg/ml Proteinase K and incubation at 55°C for 10 min. 10% glycerol was added and the DNA species were separated on a 0.8% agarose gel for 1 h at 70 V. Subsequently, the gel was stained with SYBR Gold and analyzed with a Imager (Typhoon).

## Supporting Information

Figure S1 **Embryonic lethality of isolated mutants.** Mutants were irradiated with the indicated doses and embryonic lethality was scored. Data shown represent the average number of dead embryos of five hermaphrodites ± S.D.
(EPS)

Figure S2 **YFP::LEM-3 expression pattern.** (A) The transgene *opIs383* [P*_npp-1_*::YFP::*lem-3*::3′UTR*_lem-3_*] rescues the hypersensitivity of *lem-3(op444)* mutants. Data represent the average of three experiments ± S.D. The progeny of 10 worms were analysed for each experiment. (B) Representative DIC and fluorescence images of embryos expressing YFP::LEM-3; in green: YFP::LEM-3, in red: membrane staining with the dye FM4-64.
(EPS)

Figure S3 **Complementation tests.** *lem-3(op444)* fails to complement (A) *rad-1(mn155)* and (B) *lem-3(tm3468)*. F1 embryonic lethality after irradiation with 30 Gy was quantified. Data shown represent the average embryonic lethality of the progeny of 10 hermaphrodites.
(EPS)

Figure S4 **Enrichment of Sf9-expressed MBP-LEM-3 fusion protein.** The protein, containing an N-terminal MBP-tag, was enriched over an Amylose-column. M: marker; prior inf.: prior infection; post inf.: post infection; sup.: supernatant; WT: LEM-3 wild type; F→L mutant corresponding to *lem-3(op444)*.
(EPS)

Movie S1 **Chromatin segregation in wild-type embryos after irradiation.** Wild-type hermaphrodites carrying the transgene GFP::H2B were irradiated with 30 Gy. Embryos were dissected 2 h after irradiation treatment. Images were taken every 20 sec (as described in Materials and Methods).
(RAR)

Movie S2 **Chromatin segregation defects in** ***lem-3*** **(** ***op444*** **) embryos after irradiation.** *lem-3(op444)* hermaphrodites carrying the transgene GFP::H2B were irradiated with 30 Gy. Embryos were dissected 2 h after irradiation treatment. Images were taken every 20 sec (as described in Materials and Methods).
(RAR)
